# William Palmer

**Published:** 1856-07-01

**Authors:** 


### Art. VII.—WILLIAM PALMER.

On Tuesday, the 13th November last, a mare, called *Polestar*, won the Shrewsbury handicap stakes. Her owner was Mr. John Parsons Cook. He was so elated at the result of the race, as to be deprived, for a few minutes, of speech. He was a young man, evidently of weak intellect. He had inherited a fortune of twelve or thirteen thousand pounds. This he had dissipated on the turf. His success at Shrewsbury was of great consequence to him, and he looked to the realization of his bets and stakes as a means of retrieving his shattered fortunes. Alas, for human calculations! In one short week—viz., on the Tuesday following—this unhappy young man died in severest agonies, the victim of the most remorseless and treacherous murder ever recorded in the annals of crime! He fell by the hand of a friend and boon companion.

Six months after, on the 27th of May, 1856, providentially for the ends of justice and the interests of society, the perpetrator of this foul murder was convicted and sentenced to execution; and, on the 14th of last month, he, in the presence of 50,000 persons, suffered an ignominious death upon the scaffold. We might well leave him in the hands of a merciful Saviour, satisfied that the majesty of justice has been vindicated on earth, except for some important psychological and medical considerations to which we consider it our duty, as public journalists, to direct particular attention.

The murder of Cook was only the climax to a series of criminal acts. It is indisputable, that Palmer's pecuniary embarrassments were extreme; that he had effected a policy on his brother Walter's life, the payment of which was refused by the insurance office; that he had not only forged his mother's name, but branded his wife in her early grave (and how early, *he* only knows) with the guilt of the forgery; that he robbed Cook of 1800*l*., and fraudulently laid claim to 4000*l*. against Cook's estate;—these alone form an accumulation of direct and circumstantial evidence of the utmost weight against him, apart from the particular crime for which he was arraigned. To those who are accustomed to weigh the value of evidence, there could not remain the shadow of a doubt respecting his guilt; everything spoke against him, nothing told in his favour; and, in fact, the strongest testimony against his innocence was elicited in the cross-examination of the witnesses for his defence.*

* "It is now six months since John Parsons Cook expired in agonies at the inn at Rugeley, and from that time to this the public interest has suffered no abatement. The terrible details of this case and of the two others in which suspicion was raised against the prisoner have been discussed in every household of the three kingdoms. Popular feeling was so excited in the neighbourhood of the deed that the prisoner's advisers asked, and the Crown acquiesced in, a change in the place of trial. A new Act of Parliament was passed to enable the Queen's Bench to send the matter before a metropolitan court. The postponement of the trial gave the prisoner every facility in preparing a plausible defence, even to the selection of scientific men to detail the events of their practice and to prosecute special experiments. The Crown, of its own free will, furnished the defence with all the evidence which it was intended to bring forward. Finally, six months after the commission of the crime, the Chief Justice of England and two other Judges celebrated for their experience and acuteness took their seats on the bench. A jury, not taken from among the farmers of a small country district, but selected by chance from the trading class of a population numbering 3,000,000 of souls, removed as far as possible every suspicion of unfairness. Then came a trial of extraordinary length and labour. The opening speech of the Attorney-General lasted more than four hours; his reply was nearly as long. The prisoner's counsel defended him in a speech of eight hours. The case for the prosecution lasted six days; that for the defence more than three. The summing-up of the Chief Justice commenced at the sitting of the Court on Monday, and was not concluded until yesterday afternoon. The men of highest standing in the medical profession gave their evidence for the Crown or for the prisoner. Finally, the jury, after listening with unwearied patience to the arguments and testimony of nearly twelve days, retired to consider their verdict. On their return into court the foreman pronounced the terrible word which consigns William Palmer to a murderer's doom.

"In the justice of the verdict every one who has followed these memorable proceedings must fully concur. Never was a crime more cruel, treacherous, and cold-blooded; never was it brought home by proof more cogent and irresistible. True, the evidence was circumstantial, but in some respects circumstantial evidence is the best. Where the proof of crime is assumed from the testimony of two or three who declare to its actual commission, there is room to doubt whether animosity, or a wish in the witnesses to screen themselves, may not have led to perjury for the destruction of an innocent man. But where a long series of facts, deposed to by numbers of persons unacquainted or unconnected with each other, all points to one conclusion, then there can be little doubt as to the decision. Never did testimony more various and more unshaken unite to bring home guilt than in the case of Palmer. Cook is well and in high spirits, and suddenly is affected by sickness for which no one can account. A few days afterwards he arrives in Rugeley with the

We have thus briefly recorded the history of the criminal, for the purpose of noticing the moral signs of depravity which form so striking a part of his character. He was a member of the medical profession—one of ourselves. He studied at St. Bartholomew's Hospital, under some of the most distinguished teachers of medicine, surgery, anatomy, physiology, chemistry of the day; so that, with but moderate talents and not more than ordinary attention, he could scarcely fail to be well educated in the rudiments of his profession. This was less than ten years ago, if his age be correctly stated at thirty-one. It does not appear that prisoner, and there the symptoms are repeated. He is not bilious, nor suffering from any complaint which should produce vomiting, and yet he is sick after everything that the prisoner administers. A servant in the place tastes some broth prepared at the prisoner's house, and suffers in the same manner for hours afterwards. When the unhappy man dies, antimony is found in the blood,—a fact which science pronounces conclusive of its having been administered within forty-eight hours before death. Yet no medicine containing antimony had been openly prescribed, nor is it pretended that the deceased was in the habit of taking any such drug. In fact, the defence totally evaded the question of the antimony altogether. The counsel brought witness on witness to give their speculations on tetanus, epilepsy, and convulsions, but no answer was made to the evidence which proved that Cook had vomited for days without a cause, and that after his death a poison which kills by producing vomiting had been found in his body in a state which showed it had been recently swallowed. Can we, therefore, come to any conclusion but that the prisoner, a medical man, having this drug in his possession, and knowing its effects, had used it for the purpose of producing in Cook symptoms which might be confounded with those of ordinary disease? For it is worthy of notice that it was not the interest of Palmer that his friend should die until the stakes and bets he had won were due, but that he should be ill and unable to receive them personally. Hence we find antimony used until Palmer has gained possession of large sums on Cook's account, and then, within a few hours, as soon as it became his interest that Cook should die, the first dose of strychnine is administered. Palmer's affairs, in fact, grew more desperate every day. The usurer who had him in his power was incessant in his demands for money. Palmer had forged his mother's name, the bills were due, and writs were out against both mother and son. Twenty-four hours might discover all; for, unless 450*l.* were paid immediately to Pratt, proceedings would be taken against Mrs. Palmer. Cook had won money at Shrewsbury races, and had it about him; bets were due to him in London. That money disappears, no one knows how, and as for the bets, Palmer receives them through an agent, and applies them to his own use, the day before Cook dies. Here, then, is a motive for haste. If Cook discovers that he has been robbed, if the creditors discover that Mrs. Palmer's name has been forged, Palmer may within a week stand in a felon's dock. He knows the use of strychnine. He knows that it kills 'by tetanic fixing of the respiratory muscles.' Perhaps he does not know that it causes horrible convulsions of the whole body, but thinks that the sufferer dies with merely internal spasms. If we believe the witness Newton, he buys strychnine on the Monday night, and on that night he administers pills to Cook, which are followed by tetanus. There are doubts thrown on the evidence of Newton, because he concealed, or at least did not volunteer it, until the eve of the trial. But, even supposing this young man to be capable, for no earthly reason, of swearing away the life of one who had never done him wrong, the case does not end here. Another witness, whose testimony is not disputed, swears positively to the purchase of six grains of strychnine at another shop, that of Mr. Hawkins, on the succeeding day, but a few hours before Cook's death. If ever anything was proved in a court of justice, it is the purchase of this deadly drug by William Palmer. The defence, loosely enough, shifted its ground as regards this question. First, it was that no poison had been purchased, and that he was more exposed to corruption during his studies than the rest of his fellow-pupils, many of whom have, since then, shown themselves to be well-informed and respectable practitioners.

Mr. Serjeant Shee, in his eloquent speech for the defence, quoted a letter of Palmer's to the lady who subsequently became his wife, penned by himself while he was yet a pupil, and evincing his affection for her. Some of the daily press affect to see in this letter nothing but deceit: for our parts, we own we believe every word of it, and think that he felt sincerely what he there expressed. It is one of those strokes of nature without which the likeness of the criminal would have been too darkly

Newton was perjured; then it was that the strychnine might have been wanted to kill dogs which annoyed Palmer's horses in a paddock. Neither of these assertions are supported by a jot of evidence. The testimony of Newton was not shaken; that of Roberts was not even questioned. As for the supposed purpose of the strychnine, no evidence followed the suggestion of the prisoner's counsel. The death of the deceased occurred on the evening which succeeded the last purchase. He died just as strychnine is proved to kill. The evidence of the medical witnesses for the Crown is decisive as to the improbability of his dying by any known form of disease. Mr. Curling, Dr. Todd, Sir Benjamin Brodie—all speak positively as to this point. Thus three main points of the case are made out fully,—the death of the deceased by strychnine, the purchase of the poison by the prisoner within a few hours of the death, and the prisoner's pressing motive for the destruction of his companion.

"Only on one point can there be the slightest doubt. The body is unskilfully dissected, and the stomach, with some of the other parts, is sent to Drs. Taylor and Rees. They find no strychnine. Of course, on this the whole defence rests. A number of medical men are brought to declare that if strychnine had been taken it must, in their opinion, be found. But one fact is worth any number of opinions. Drs. Taylor and Rees perform experiments with rabbits, giving them not large doses, like the defence doctors, but just enough to kill. In two cases strychnine is found; in two it is not. Therefore these two gentlemen are justified in declaring that, according to the tests made use of by them in the case of Cook and in the case of the animals, the poison is sometimes found and sometimes not. We cannot but think that the witnesses for the defence endeavoured to prove too much. Scientific dogmatism could go no further than when two gentlemen alleged that Cook could not have taken strychnine because he allowed himself to be touched,—an act which always threw a rabbit into a spasm. Equally unavailing for the prisoner were the suggestions as to the cause of death; it was apoplexy, epilepsy, idiopathic tetanus, traumatic tetanus, epilepsy with tetanic complications, and so on. There were as many opinions as men; and though certainly a prisoner is not bound to account for the cause of death, yet a jury, observing the differences between these witnesses, who made such a display of science, might naturally be led to think that their opinions were not sufficiently authoritative to destroy the testimony of facts and the deductions of common sense. If we add to this that in one case a medical witness confessed to having expressed a belief in Palmer's guilt, and an opinion of the incompetency of Dr. Taylor to detect it, we can have little wonder that the jury should have made so little of the large array of testimony for the defence.

All these points were fully noticed by the Chief Justice in his long and conscientious summing up, as well as those minor incidents which strengthen into certainty the belief of the prisoner's guilt. The anxiety about the jar, the presents to the coroner, the attempted bribing of the postmaster and the postboy, the curiosity about Dr. Taylor's analysis, leading the prisoner to procure the tampering with his letter, and inconsistent with the knowledge that no strychnine had been administered, all forced home the conclusion of the prisoner's guilt."—*The Times*, Wednesday, May 28, 1856.

coloured and deformed; and if, in spite of this early avowal, he afterwards ended by maligning, if nothing worse than maligning, the first object of his affections, it is only one proof more of the desperate means that a course of vice forces us to at last—*Nemo repente turpissimus fuit.**

He was essentially selfish—it is the main feature of his character. Everything turned upon SELF. The murder of Cook, the alleged murder of Walter Palmer, his brother, his design upon Bates, his treaty with Pratt for the possession of the winning horse, *Polestar*,—all turned upon self. Everything was made subservient to this end; he was ready to sacrifice the world to it; he did, in fact, sacrifice his own mother to his unworthy purposes; and, in endeavouring to suborn the postboy, he was, at the same time, ready to sacrifice Mr. Stevens's safety, by the upsetting of the fly, in order to break the jar which, in all probability, contained the damning proof of his own culpability.†

* "The mass of the evidence against him may be broadly ranged under two principal heads. 1. The moral. 2. The scientific. As regards the moral, or circumstantial branch of the evidence, the most zealous opponents of the propriety of the conviction would probably admit that, taken by itself, it enormously preponderated against the prisoner. We need not again travel over ground with which the general public are so familiar. Suffice it to say, that the following are a few of the points which the prisoner's defence left wholly untouched:—No answer was given to the charge that Palmer had, within a few days before Cook's death, applied nearly 2000*l.* of Cook's winnings to satisfy the claims of his own money-lenders; no account was given of the disposal of the strychnine which Palmer was proved to have purchased on the very eve of Cook's death; no explanation was given of Palmer's strange eagerness to get the body put into a coffin, of his demeanour at the *post-mortem* examination, of his tampering with the jars containing the matter about to be sent up to Dr. Taylor for analysis, of his attempt to bribe the postboy, of his inquiries as to the possibility of discovering strychnia in the body, of his exultation on receiving a negative answer, of his eagerness to ascertain the result of Dr. Taylor's report, and his subsequent endeavours to pervert and misdirect the mind of the coroner. But on these and on other similar points it is really unnecessary to dwell. As far as the circumstantial evidence is concerned, all those who by patient attention to the case have qualified themselves to form a judgment, must admit that, in its cumulative effect, it told with overwhelming weight against any rational theory of the prisoner's innocence."—*The Express*, June 7, 1856.

† "The very moment when he was enjoying the good-will of his neighbours, living apparently an easy, careless life of enjoyment, he was inwardly distracted by all the passions of the gamester. Under the jovial expression of these rubicund cheeks, who could have imagined the secret history which was being transacted? For this jolly fellow was racing, betting, winning—more often losing—and encumbered with debt. To relieve himself from his obligations he was contracting more debt—selling himself body and soul to money-lenders—using the hand of his own wife (he himself confessed it) to forge the name of his own mother on bills of acceptance for thousands of pounds, knowing at the very time that nothing but a lucky cast could make him to discharge those obligations, which, nevertheless, if not discharged, must prove passports to prison. Nor was this all. Other means were needed to satisfy his wants: and Palmer was not the man to hesitate. He had a wife and a brother. Money must be had. He insured their lives. His brother was fond of liquor. He hired a wretch to lead him on to fatal excess, and death soon overtook

Let us analyse the character still more closely. There were some vices from which he was singularly free. He was not proud, but, on the contrary, he stood greatly in need of so superb a passion as that of pride, which might have preserved him from his other more desperate and debased propensities,—it would have imparted to him a higher bearing, and the appearance, at least, of nobler behaviour; only, he was not proud. Nor does he seem to have been of an angry and choleric temperament, but just the reverse—always cool and collected, wary and sagacious. Neither was he gluttonous nor drunken; and even his more sensual pleasures he managed with so much address, that they did not disgrace his reputation so much as they ought to have done. He could join his friends at the table, but he only lent himself to it, without suffering it to take the mastery over him, or to deprive him of his senses and vigilance. He was too cautious to be caught in his own trap. And, lastly, he was not slothful, but most diligent in the object of his research; for he left nothing unattended to, albeit his engagements ultimately outstripped him in the race.

"Consider the man. In the eyes of his fellow-townsmen this country surgeon had acquired a character for respectability. To most people he seemed an agreeable person—to many even gentlemanlike. He lived in his native town of Rugeley; he was admitted to its leading official society. He had soothed the pangs of many a youthful mother, and watched the sick-bed of many a first-born infant. Latterly, indeed, he had withdrawn from the practice of his profession, having betaken himself to Tattersall's and the turf.

"In the character of William Palmer there is, indeed, a sort of dramatic monstrosity. He was no common-place villain. The bludgeon and the horse-pistol—the knife and the centre-bit—were not his weapons. Like Cæsar or Napoleon, his pawns were men. His instruments were mortal. He turned everything to his own use—his personal appearance—his professional knowledge—his mother—his brother—his school companions—his friends—the friends of his friends—postmasters — money-lenders — coroners—profligate attorneys."—*The Leader*.

His sense of justice, or probity, was very small. He squanhim. His wife was his slave—to be coined into cash. As a medical man he might himself minister to her ailments. She must die. Her husband—so will the legend for ever run—will himself conduct her to the gates of death. He seats himself by her bedside—with his own hand he tenderly administers the poisonous drug—writes down his grief in his private diary—watches the vital power of the poor creature slowly evaporate under his fatal skill—consoles his wounded spirit for her fortunate loss at the very steps of the altar—and then gathers in the golden harvest of deliberate crime. This surely is not the man who would reveal himself to the thousand eyes of a crowded court of justice. He had played the stakes of life and death too often and too successfully before he stood within that dock. In the last momentous scene—the crisis of his life—no weak emotion was to disturb the serene apathy of this consummate artist."—*The Leader*, June 7, 1856.

dered his own means, unscrupulously made use of those of others, and disregarded truth, life, honour, and money alike. He carefully removed every obstacle that stood in his way, and made use of every means in his power to facilitate his plans. He killed Cook, forged his mother's signature, rifled his associate's purse the moment the breath was out of his body. He was a liar of the first magnitude. His last instructions to Pratt, the solicitor, were most of them false ; his answers to Mr. Stevens respecting the betting-book were prevarications too transparent not to be seen through ; his hints as to what they were likely to find in the *post-mortem* examination of Cook's body ; his telling Mr. Jones that Cook was suffering from a bilious attack ; and his persuading Dr. Bamford to fill up the certificate of death with the word "*apoplexy*," were all of them deliberate lies. The theme of his life was lying and money-getting throughout. Medicine offered no opportunity of making a fortune in any way equal to that of betting, racing, and life-insuring. He might, in the course of a long career, have realized a few thousands by means of the practice of medicine ; but this was much too slow a game for one whose love of money could only be gratified with thousands and tens of thousands, though lost as soon as gained, and scarcely enjoyed even when won.

"He possessed," says the *Daily News*, "every requisite which would, *a priori*, be deemed necessary to success in the career to which he devoted himself; and yet this cold-blooded schemer, who never allowed any considerations of wrong, or morality, or fear, to stand for a moment between his desire and its object,—this calculator who had reduced life to a game of chance,—money his only object, and crime his familiar means,—was singularly unfortunate as a speculator on the turf. The insurance of his wife's life—say, rather, the blood-money received for his wife's murder—paid off with difficulty the racing debts of 1854. In 1855 those debts had again reached the enormous aggregate of from 10,000*l*. to 20,000*l*. And yet the die with which this man played was loaded,—the hand that threw it never trembled with compunction. He owned horses, he was intimate with trainers, he was deeply versed in all the secrets of the turf; and yet this was the result—ruin, forgery, murder, and, finally, the scaffold. We disclaim the puerile exaggeration which would represent even the professional turfman as necessarily a candidate for ruin, or an adept at crime. Neither would we be so unjust as to hold up to reprobation the men in high station who actively engage in such sports. A great distinction should be drawn between those who engage in the turf as a mere gaming speculation, and he who is influenced by right and honourable motives—viz., the improvement in the breed of horses. We must, however, admit that a life whose business is reckless and illegitimate speculation, whether on the racing-course or at the gaming-table, or in the Stock Exchange, is a life of all others most demoralizing in its tendencies, and is obviously unfavourable to the healthful development and training of the mental and moral faculties. It is a career in which occasional success establishes nothing beyond the copy-book truism, 'that there is no rule without its exception,'—a career in which, for one prize, there are a thousand blanks,—a career in which the most brilliant success is less enviable than success in any other field of legitimate enterprise,—a career in which failure has recently led to such crimes as those by which Sadleir has lately ruined a province, or that more execrable guilt which, in Palmer's case, has appalled a world."

Another chief quality was his self-possession.

"His self-command was perfect. His readiness of resource marvellous. His presence of mind never forsook him. In powers of far-reaching combination he has rarely been surpassed. In the still more singular gift of making absolute tools of those whom he wished to use, the annals of greatness or villany might be searched in vain to find his peer. In this respect Jonathan Wild the Great was nothing to him. The fancies of Fielding's fiction are distanced by the facts of Palmer's history. Cheshire, the postmaster, was his thrall, Cook his cully, old Dr. Bamford his dupe; he won over Ward, the coroner, by his presents;—nay, even in the supreme crisis of his fate, he contrived, apparently, to produce a belief in certain quarters that the righteous sentence just passed upon him was rather the result of popular prejudice than of even-handed justice."*

* "The evidence of one witness on the trial of William Palmer is of especial interest to the community, and, as any comment upon it can have no effect upon the case, we call attention to it at once. The pecuniary troubles of the prisoner for many months previous to the death of his companion, Cook, are detailed at great length by Mr. Thomas Pratt, solicitor, of Queen-street, Mayfair. The history is instructive for those who may at any time feel inclined to begin the seductive practice of borrowing money on their own personal security. It certainly requires no great arithmetical genius to calculate what 1000*l.* at 60 per cent., payable monthly, will amount to in a given term of years, nor is there much forethought necessary to show a man that, with the most unceasing and fortunate exertions, he is hardly likely to extricate himself from debt which increases with such terrible rapidity. The evidence of Mr. Pratt is an old tale, and often told,—the first small loan duly repaid, then a larger sum, then the borrower's acceptances, then those presumed to be his mother's bills renewed, interest payable monthly, threatening letters, entreaties for delay, falsehood, forgery, appropriation of another man's money, and then the final crash. It is worthy of attention in all its naked simplicity.

"About two years and a half since the acquaintance between Palmer and Mr. Pratt began. Palmer borrowed 1000*l.*, which he repaid. More seems to have been borrowed during the ensuing year from Pratt or other persons, for when Anne Palmer, the prisoner's wife, died, and Mr. Pratt received 8000*l.* from the offices on account of the policies on her life, 6500*l.* was at once applied in payment of bills due. In April, 1855, Mr. Pratt was again applied to for a loan of 2000*l.* on a bill purporting to be accepted by Mrs. Sarah Palmer, and by November last there were eight bills held by Mr. Pratt or his clients, the total amount being 12,500*l.* 'The interest,' says the witness, 'was paid monthly. With two exceptions, these bills were discounted at the rate of 60 per cent.' Thus the interest to be paid by Palmer for money borrowed within seven months was probably upwards of 6000*l.* a-year, the income of a leading barrister or physician. If the prisoner had become possessed of an annuity equal to the salary of a judge or a minister of state, it must have been swept away yearly during his whole life in merely paying

The seeming nonchalance and indifference with which he listened to the sentence of the judge and quitted the dock, is the interest of this temporary 'accommodation.' No wonder there are soon urgent letters on the part of Mr. Pratt, and evasive answers from his client. Walter Palmer is dead, but, even if the Prince of Wales Insurance-Office will pay the 13,000*l.*, it cannot be received directly; so Mr. Pratt writes, 'Do pray think about your three bills so shortly becoming due. If I do not get a positive appointment from the office to pay, which I do not expect, you must be prepared to meet them, as agreed. You told me your mother was coming up this month, and would settle them.' Again, on the 2nd of October, 'Bear in mind that you must be prepared to cover your mother's acceptances due at the end of the month.' On the 18th, 'I send copies of two letters I have received. As regards the first, it shows how important it is that you or your mother should prepare for the payment of the 4000*l.* due in a few days.' What is the position, then, of Palmer? He has 4000*l.* to pay, and can only pay 250*l.*, and promise 250*l.* more. 'For goodness sake, do not think of writs. . . . . I will get you the money, even if I pay 1000*l.* for it.' The two sums of 250*l.* seem to have mollified Mr. Pratt for a few days. On the 31st of October he writes—'The 250*l.* in registered letter duly received to-day. With it I have been enabled to obtain consent to the following.' And then he tells Palmer that, with the exception of issuing writs against his mother, no proceeding as to service shall be made until the morning of Saturday, the 10th, 'when you are to send up the 1000*l.* or 1500*l.* *You will be debited with a month's interest of the whole* 400*l.* *out of the money sent up.*' On the 11th of November Palmer was not ready with the 1000*l.*, but he brought 300*l.*, making in all 800*l.* paid;—'200*l.* *was deducted for interest, leaving* 600*l.*' Thus, out of the money which Palmer had scraped together in the course of the past month, a fourth—200*l.*—went as the month's interest of the 4000*l.* Of course Mr. Pratt was still pressing for his balance of 3400*l.* On the 17th Palmer is able to send a check for 200*l.*; on the 19th he pays in 50*l.*, and promises 450*l.*, which accordingly was liquidated out of Cook's money. But even then the call was for more. On the very day of Cook's death Palmer writes, 'I will send you the 75*l.* to-morrow.' This 75*l.*, it afterwards appears, was for the renewal of a bill for 1500*l.* for one month, being at the same reasonable rate of 60 per cent. On the 24th of November Palmer paid as small a sum as 25*l.* on account of a 2000*l.* bill due a month before.

"Such are the transactions at this present day between borrower and lender. A man involves himself in debt, of which the interest every eighteen months equals the principal, and another is found to lend him the money, with, we must presume, a knowledge that he has no settled occupation, and that his resources are only derived from the betting-ring, or contingent on what he may inherit from his relatives. These are not transactions between a youth at the University and a wine-merchant acquainted with his prospects. They seem the ordinary dealings between two grown-up men, each of whom has had considerable experience of the world, and especially of that shrewd class with which Cook and Palmer associated. One man forges his mother's name, and pays 60 per cent. to have the bills renewed; the other takes his 60 per cent., which Mrs. Palmer is supposed to allow her son to pay for the accommodation. It cannot be doubted that, although commercial pursuits are highly honourable, such a trade as that of Mr. Pratt and his 'clients,' of whom he 'obtains consent' when a bill is to be renewed, is hardly calculated to improve the public morality. The wonder to a reflecting man is, how any one should give way to such madness as to involve himself in meshes from which he must in reason be convinced there is no escape. The sudden pressure of some 'debt of honour' leads, probably, to the first application, and once fairly entangled, there is no escape. One who trusts so much to fortune as the betting man, no doubt thinks that the next 'event' will bring deliverance from all his troubles, and first in confidence, and at last in desperation, he continues his terrible course. It may be beyond the power of law to prevent such dealings, but we may certainly say that the man who supplies money to one thus rushing to ruin deserves any loss he may sustain, and that anything which may lessen the gainfulness of his trade will be a benefit to society."—*The Times*, May 22, 1856.

nothing more than vulgar and impudent bravado, and if it proves anything, proves too much for his cause. Under such awful circumstances, an innocent prisoner would be ready to sink into the earth with shame and confusion—the mere suspicion would be enough to harrow his feelings and congeal his blood. But to bear—not the suspicion—not the imputation—but the very conviction of crime, in the open face of day, with composure—to speak of it with philosophic indifference, and to declare that it will not even so much as disturb the night's repose—this is the sign of hardened villany and a callous conscience, unless it be sheer affectation and practised effrontery. Palmer's allusion to God as the defender of innocence, can only be viewed as another part of the same detestable jargon.*

* *The Express*, of June 2, says Thurtell confessed a dozen murders which he certainly never committed. He declared that he drowned a young man at Norwich who was known by the townspeople to be non-existent; and he told ridiculous stories of knocking gamblers on the head with dumb bells behind doors in gaming-houses; where nothing of the sort ever took place. This sort of bravado, and that with which he went out of the world, produced something of the effect he intended on ignorant and impressible minds, and a bad tone was introduced into the immorality of young scamps of that day which aggravated the grief they caused at home and the mischief which they diffused abroad. When the murderer is not a hero he tries hard to be a saint; and as the lower part of the press has been usually more or less to blame in the first case, the weaker part of the religious world have been so in the other. Since Dr. Dodd—who fully expected to be in heaven before Dr. Johnson—there have been plenty of wretches who have regarded, or pretended to regard, the drop as the gate of heaven. Public good sense has of late years so risen up against this folly and blasphemy that we may hope it has been checked for a time.

### THURTELL AND PALMER.

With the difference that the circumstances of Thurtell's guilt are not comparable in atrocity with those of the poisoner's, there are points of strong resemblance between the two men. Each was born in a fair station, and educated in conformity with it; each murdered a man with whom he had been on terms of intimate association, and for whom he professed a friendship at the time of the murder; both were members of that vermin race of outer betters and blacklegs, of whom some worthy samples were presented on both trials, and of whom, as a community, mankind would be blessedly rid, if they could all be, once and for ever, knocked on the head at a blow. Thurtell's demeanour was exactly that of the poisoner's. We have referred to the newspapers of his time in aid of our previous knowledge of the case, and they present a complete confirmation of the simple fact for which we contend. From day to day during his imprisonment before his trial he is described as "collected and resolute in his demeanour," as "rather mild and conciliatory in his address," as being visited by "friends whom he receives with cheerfulness," as "remaining firm and unmoved," as "increasing in confidence as the day which is to decide his fate draws nigh," as "speaking of the favourable result of the trial with his usual confidence." On his trial he looks "particularly well and healthy." His attention and composure are considered as wonderful as the poisoner's; he writes notes as the poisoner did; he watches the case with the same cool eye; he "retains that firmness for which, from the moment of his apprehension, he has been distinguished;" he "carefully assorts his papers on a desk near him;" he is (in this being singular) his own orator, and makes a speech in the manner of Edmund Kean, on the whole not very unlike that of the leading counsel for the poisoner, concluding, as to his own innocence, with a So help me God! Before his trial the poisoner says he will be at the coming race for the Derby. Before his trial Thurtell says, "that after

We find the same mental phenomenon in nearly every phase of crime. The expression of strong religious sentiment is often common to great culprits. It is difficult to discriminate between their sincerity, however ill-timed, and their hypocrisy, however useless, when employed for the sake of glossing over their known and conscious iniquity. It may be both the one and the other; but whether or not, none except the weak-minded and inexperienced is ever deceived by it. It sounds like what it is, a disgusting blasphemy. William Palmer, it is said, went to church and received the sacrament the Sunday after his (murdered ?) wife's decease:—

> "With devotional visage
> And pious action, we do sugar o'er
> The devil himself."

Viewed psychologically, such mental phenomena are to be considered. Either it is an impairment, vitiation, or total want of the moral sense, mostly connected with debility, or struma, or disease of the nervous structures, sometimes connected with drinking, or severe abstinence, or reverie, or preternatural energy, or the loss of self-control, to a degree which none, except those acquainted with the natural history of mania, have the most distant notion of. It is the same as the hump-back, or the club-foot, or tuberculosis, in common pathology, and an outstanding symptom of a disordered intellect. It is shocking to see religion thus travestied; but, then, the best things are the most abused—fictitious piety is an excellent mask for wicked purposes. True religion is never ostentatious, nor does it court popularity. It hides itself within the heart, and is practically known by the life and conduct of those under its influence.

Palmer was what the *Times* characterized him—a most "vulgar criminal." To our taste, his gait, his physiognomy, his *chevelure*, and his attire, were essentially vulgar—not the vulhis acquittal he will visit his father, and will propose to him to advance the portion which he intended for him, upon which he will reside abroad." So Mr. Manning observed, under similar circumstances, that when all that nonsense was over, and the thing wound up, he had an idea of establishing himself in the West Indies. When the poisoner's trial is yet to last another day or so, he enjoys his half-pound of steak and his tea, wishes his best friends may sleep as he does, and fears the grave "no more than his bed." (See the "Evening Hymn for a Young Child.") When Thurtell's trial is yet to last another day or so, he takes his cold meat, tea, and coffee, and "enjoys himself with great comfort;" also on the morning of his execution, he awakes from as innocent a slumber as the poisoner's, declaring that he has had an excellent night, and that he hasn't dreamed "about this business." Whether the parallel will hold to the last, as to "feeling very well and very comfortable," as to "the firm step and perfect calmness," as to "the manliness and correctness of his general conduct," as to "the countenance unchanged by the awfulness of the situation"—not to say as to bowing to a friend from the scaffold "in a friendly but dignified manner"—our readers will know for themselves when we know too.—*Household Words.*

garity of untutored ignorance and rusticity, but the saucy, jaunty, and confident vulgarity of low life.*

Palmer had not the physiognomy of a great criminal. There was nothing positively forbidding or repulsive in his countenance. His anterior cranial development was good—the *posterior* part of the head (the seat of the animal passions, according to phrenologists) was obviously large. He had a thick and coarse neck. Considering Palmer as a physiognomist, no person could have said—

> "*This is the man should do the bloody deed,*
> *The image of a heinous fault*
> *Lives in his eye; that close aspect of his*
> *Does shew the mood of a much troubled breast.*"

or that on

> "*His eye-balls murdered tyranny*
> *Sat in grim majesty to fright the world.*"

He looked the good-natured, easy, well-spoken, and jolly tavern companion, always ready to seize a friend by the hand, and to join a boon companion in the festive cup. So much for his physiognomy.

And here we take leave of Palmer as a psychological phenomenon, and may we never look upon his like again. Let us turn to the medical evidence, and consider its nature and utility on an occasion so trying as this has been. The medical evidence, as it is called, divides itself into the medical and chemical; and of these two, the chemical is subsidiary to the medical; for, without the physician to judge of the symptoms, the chemist is not competent to decide upon the cause of death. It is all very well to send portions of a dead body up to London, for the first analytic chemist of the metropolis to test by its proper chemical re-agents, and then for him to declare that he finds such and such a poison in them, to the very fraction of a grain, and that this poison was the cause of death; it is all very well and very scientific for the chemist to be able and to be allowed to make this declaration, nor have we any ground for supposing that he does not declare the truth;—but, after all, what does it amount to? What is the *bonâ fide* result of the analysis? Does it, in fact, absolutely account for the death? Is the final test equal to the emergency? We maintain not.† We affirm that these

* As an instance of Palmer's self-possession and coolness during the trial, it may be mentioned that he was continually writing notes commenting upon the evidence, and giving instructions. The following is one of them. Alluding to one of his medical witnesses, he wrote to his attorney or counsel, "This is a muff, and is doing us more harm than good."

† The verdict of "guilty" which the jury has given, if uninfluenced, as we may well suppose it to be, by the pathos of the defence or the declamation of the proquestions can be answered only by the physician who was in attendance, and who dispassionately considers the individual symptoms of each case brought under his notice. But, nay, we are answered ; the chemist did, in fact, find the arsenic—here it is ! Granted : but did the patient die with the symptoms of poisoning from arsenic, or mercury, or whatever else it might be that the chemist had succeeded in detecting in the remains subjected to his chemical experiments ? In short, did the arsenic, &c., kill him ? He might have taken the arsenic, and the arsenic may have been found in him, and yet he may not have died from the effects of arsenic as a deadly poison. Did he then die from the effects of the arsenic found ? This is the question : not what was taken, nor what was found, but whether what was found and taken was the cause of death ? The physician alone can answer this question—not the chemist. We know a lady at this moment who is always, and always has been taking mercury, with or without medical advice. Now, if when she dies, her mortal remains were to be enclosed in a jar and sent to Dr. Taylor for analysis, doubtless he would detect no small quantity of mercury in all her tissues. Yet she has not died, is not dying, from the effects of the mercury. Every medical practitioner knows instances of persons who are in the habit of taking as much opium daily as would, at the first dose, kill another not habituated to the use of the drug in such secution, is a verdict which must be respected, to whatsoever consequence it may lead. We will not, in this place, attempt to review or detail the nature of the evidence adduced on both sides, and upon which the jury were required to decide. Circumstantial evidence never became more elaborate or more lengthy, and the serious question that arises for the future will be, how far evidence of that nature, involving scientific difficulties, can be regarded as the foundation of a death penalty ? But there is another question, which at this moment, we should not seek to avoid. The prejudice in the public mind against the prisoner was so far just that it had its origin in the natural detestation which is felt against great crimes. It was assumed that he had become an adept in the art of poisoning his friends—that his nearest relatives and closest companions were not safe from his power—and that no language could be strong enough to describe the cold, savage, and diabolical skill with which he had, more than once, executed his purpose. Now, if all this, and even much more, instead of one act of homicide, had been proved, we should be asked, if it would be wise and safe to abrogate the capital penalty, when so much outrage is by one man committed against the feelings of society ? Must there be no opportunity for the strongest evidence of the public detestation —DEATH ?

We think not. Let it not be imagined we believe that William Palmer is a greater criminal than the jury have made him out to be ; far less let it be imputed to us that we wish to palliate tragedy of any form or degree. But the answer to the difficulty which this case has apparently raised is either an inspired answer or a human one. If inspired it will stand thus : "Vengeance is Mine." If human, it could not be better put than in the words of a distinguished member of the French National Convention who, before he became corrupted, said, "To kill a man—know ye what that means ?" "It means," he continued, "to kill his possible return to virtue, to kill expiation, and—infamous act—to kill repentance itself !"—*Morning Star*, May 28, 1856.

quantities. Were such a person to die, say from asphyxia,* soon after he had swallowed his last quotum, opium would doubtless be found in his stomach in a deadly dose: and yet the opium would not be the cause of the death. Again; persons are in the habit of drinking wine or spirits very freely, all their lives, and live to be old in spite of their excess: of course, if such a one dropped suddenly from some accidental cause, not visible externally, ardent spirits might be found in the ventricles of his brain even, without their having been the cause of his decease. We knew a gentleman who was affected with rupia, a skin disease, on his face. He consulted a medical practitioner, who cured it by arsenic. Subsequently this gentleman took arsenic by himself, whenever the disease reappeared, and always with the best effects, and we believe he still continues the same practice whenever the symptoms recur: now, though this gentleman were to die, and his viscera, &c., submitted to the proper chemical re-agents, and arsenic found, as it needs would be, in every part, yet the arsenic could not, *per se*, be pronounced to be the cause of death; because it has not only not killed him, and years have

* Difficulty of Detecting Poison in the Blood.—At an inquest held on Wednesday, by Mr. Wakley, coroner for West Middlesex, on a child poisoned by the impure air inhaled under the bed-clothes— a circumstance by which the coroner remarked at least 150 children annually lost their lives in his district of the county alone—Mr. Wakley, after the inquiry had been gone through, commented upon the recent mysterious facts in connexion with the trial of Palmer, observing—"A good deal has lately been said about poison in the blood and chemical analysis. Now, this child died, literally speaking, from poison; but, if a careful analysis of the blood was to take place, not the most shrewd man could say what poison it contained. The child is covered over by the bed-clothes, it goes on sleeping, the blood becomes poisoned from the want of natural air, and, eventually, life stops, the departure of vitality being similar to the slow flickering of an extinguishing candle, when it has no wax or tallow to support its further burning. I have had as many as seven such cases in this parish during one week, and it is the most difficult—indeed, an impossible thing—to prove whether the children had been wilfully so poisoned or not—(Sensation). Not all the science in the world could prove if the deaths, in many cases, were accidental, or brought about by design."

"A case is on record where a man was in the habit of taking 3½ grain doses of morphia for tic doloureux. By mistake the druggist gave him strychnia. He remarked at the time he took it that it was very bitter. While walking along the street afterwards he felt numbness along the back of his legs. He went by a public conveyance to a village where his business lay, and returned by the next opportunity, experiencing all the time the same symptoms. On his return they became worse, "accompanied by a sense of want of power, and a sort of dragging of the muscles of the legs, which soon became so great, that, as he describes it, he had to put his hands at the back of his thighs in order to push his legs along." This was two-and-a-half hours after swallowing the supposed medicine. There was so little unusual in his appearance, that a friend laughed at him. While showing the effect to his friend, and bending himself, he suddenly fell, and on being raised felt excessively nervous and alarmed. His friend saw him home. He went to bed five hours after having taken the poison. He then took a similar dose, and in ten minutes was seized with violent spasms of tetanus, which recurred at intervals of a quarter of an hour. In thirteen hours he recovered, having taken seven grains of strychnia!"—Dr. Glover: *The Express*, May 28, 1856.

elapsed since he first began taking it, but it has, on the contrary, been the means of restoring and preserving his health. So that the mere chemical evidence of poison being found in the body is no proof, either absolute or presumptive, of the person having died from the effects of the poison so found; and to conclude that the poison found by the chemist is the real cause of the death, would lead to very grave and dangerous consequences. The physician who *saw* the patient last is the only person who can say whether the poison found was or was not really, or likely to be, the sole and absolute cause of the patient's decease.

Too much stress is laid on the chemical evidence, although the final decision of the jury did not, in the recent trial, turn upon it, good as the chemical evidence might be. The moral evidence was more than enough. But before chemical evidence can ever be relied on in a court of law, we must have a chemical board, publicly appointed and legitimately authorized, to make these chemical experiments; and, for the future, no single man, nor even several men, however eminent their position or undoubted their skill, should be intrusted with such important and delicate investigations. Should not the accused person be represented at this chemical board? *We* would not trust our lives in any analytic chemist's hands, whoever he might be; and, in truth, if we were on the jury, we would never find a verdict of wilful murder against a person upon the sole *ipse dixit of one analytic chemist, who secretly performed his investigations in his private laboratory, and then alleged that he had, after boiling down a man's liver, lungs, spleen, and heart, detected the* 50,000*th part of a grain, or even half a grain, of arsenic, strychnia, or antimony.* The idea is monstrous; the precedent, if allowed, pernicious to the last degree; and no life is safe under such an irresponsible mode of proceeding. These delicate investigations should be in the hands, not of a *private chemist who may be retained on either side, but of a public, acknowledged, and responsible board of independent and skilful men, whose minds cannot be prejudiced one way or the other.*

But to return to the consideration of Palmer's case. The circumstantial and moral evidence* was strong, convincing, un-

* "Palmer has had the benefit of such a trial as we believe he could not have obtained in any other country whatever. It is with great satisfaction that we compare the method of procedure observed in this case with such a trial as that of the Duke de Praslin or of Madame Laffarge. No traps were laid for the prisoner —he was not entangled in his own admissions, nor convicted by his own confusion. His guilt, on the contrary, was demonstrated by a clear chain of reasoning, which inevitably connected him with the crime. Mr. Smith's endeavour is to show that he was condemned upon what is called chymical evidence alone. He tells us that the theory of absorption as propounded by Dr. Taylor is new and hypothetical, and not in any way warranted by experience. He adds, 'If strychnia is not absorbed and decomposed, and can be found under similar circumstances to those which answerable, and conclusive; but not so, according to our judgment, the legal. Palmer was indicted for murdering John Parsons Cook by means of strychnine; and strychnine was proved to have been purchased by Palmer beyond a doubt; but there was no positive proof that Palmer administered it to Cook, or that Cook died from its effects, except the symptoms of death, which were alleged to result from strychnine poison—no strychnine having been detected in the body! As to Dr. Taylor's theory, respecting which so much has been written, to our minds it was wildly hypothetical and vaguely speculative. He found himself in a difficulty, and that difficulty was his apparent defective analytical investigation of the contents of Cook's stomach. In Taylor's report, he says that Cook died, "in absence of natural causes of death, of antimony," he having discovered half a grain of it in Cook's liver! Dr. Taylor was forced to admit that that quantity of antimony was insufficient to account for Cook's death, and, when pressed upon the point, said that Cook must have taken more than that. Why? *Credat Judæus,* because it passed off by vomiting! Hypothesis again! But why "passed off?" Is such the fact?* If sufficient tartar emetic be given to excite sickness, what evidence have we that it "passes off" at all in the substance vomited? Does it not first *enter the blood,*

existed, and now exist, in the case of the late John Parsons Cook, then my client will have been the victim of an erroneous conviction, if strychnia cannot now be discovered in the body of the deceased.' Now, this is a view of the case in which we cannot acquiesce. Even if Professor Taylor's theory with regard to the absorption of strychnine be proved by subsequent experiments to be erroneous, enough appeared at the trial not only to warrant, but to necessitate, the condemnation of Palmer. The only conclusion we draw from Mr. Smith's remarks is, that if the absorption theory be incorrect, then the manipulations of Professor Taylor were unskilfully performed. Whether he could discover it or not, the strychnine was there. Mr. Smith should remember that the chymical evidence with regard to the presence or absence of strychnine was one thing, the medical symptoms quite another."—*The Times,* June 5, 1856.

* STRYCHNINE—ITS EFFECTS ON ANIMAL LIFE.

*To the Editor of the Times.*

"Sir,—As some of the most eminent toxicologists seem to disagree relative to the effects of strychnine when taken into the animal economy, perhaps the following facts may be interesting to your readers.

"Being in Mexico in 1849, I had an opportunity offered me of witnessing the wholesale poisoning of wolves by nux vomica. The plan adopted is to give an old worn-out mule the poison. In a short time death ensues; the wolves, which infest that part of Mexico—Parras—devour the carcase; they after a few hours die; their bodies in turn are eaten by the turkey-buzzards, who also die.

"These facts demonstrate that the active principle—*i.e.* strychnine—is not destroyed by absorption, but remains undecomposed, though disseminated throughout the system in the most infinitesimal atoms.

"Yours,

"June 3. Philo-Veritas."

*The Times,* June 5, 1856.

"If it is worth while to have a police—to have judges and lawyers, it is worth while to provide some adequate endowment or inducement for the cultivation of and subsequently act upon capillaries of the stomach, inducing sickness and vomiting? When speaking of the *modus operandi* of the tartrate of antimony, Dr. Headland observes,—

"By an influence on a part of the nervous system—apparently the vagus nerve—it produces, first, the state called nausea, and afterwards, vomiting.

"This nausea is not produced to any extent by a mere irritant emetic, such as sulphate of zinc, which acts externally and takes effect immediately. The antimonial cannot act so quickly; part of it must first be absorbed, so that it may reach the nerve. We know that it does not act by outward irritation, from the fact that if the solution be injected into the veins at any part of the body, it will equally produce nausea and vomiting."—*On the Action of Medicines*, by F. W. Headland, M.D.

Again, Dr. Taylor says, the *minimum* dose of strychnia cannot be detected, but the *maximum* can. Well, admit this hypothesis: what proof was there that the *minimum* dose was given by Palmer? From the severity of the symptoms that preceded Cook's death, we should infer (if they arose from strychnine) that Palmer had administered to him, not the *minimum*, but the *maximum* dose. Again, we say that, strong as the moral and circumstantial evidence was, the strictly legal evidence was most unsatisfactory and defective.

How much public anxiety, expense, angry altercation, professional bickering, and disruption to healthy, calm, and tranquil thought, would have been saved, had the presence of strychnine poison been detected by Dr. Taylor when he made, in the first instance, the chemical analysis of the contents of poor Cook's stomach! It is alleged by some of the most able toxicologists and chemists of the day, that if Cook's death had been caused by the minutest dose of strychnine, Taylor *ought* to have discovered it. They assert that his mode of analysis *must* have been defective. So say Messrs. Herapath, Letheby, Nunneley, Rodgers; and the latter gentleman says, in a letter published in the *Times* since Palmer's conviction,—

"I cannot conceive an opinion more dangerous to public safety than that a fatal dose of poison can be so nicely adjusted as to escape discovery after death. Yet such has obviously been the tendency of many letters published in various journals for some time past. It was with feelings of deep regret that I noticed in your edition of to-day a medical jurisprudence. A medical witness is not an ordinary witness—he is an investigator. On the strength of his professional skill depends the value of his testimony; and as the labourer is worthy of his hire, the attention of the public is seriously called to the importance of a subject connected closely with the safety of property and of life."—*Dr. R. M. Glover, F.R.S.E.—The Express*, May 28, 1856.

communication from a former colleague of mine, Mr. Ancell, who I am sure would never have sent it, had he been aware of the nature and results of numerous experiments lately made by myself independently, and in conjunction with Mr. Girdwood. I have asserted, and do assert, that strychnine cannot evade detection if proper processes be employed for its separation.

"In this opinion I am supported by the highest chemical authorities of the day, and now request a space in your valuable columns to give to the world a process which accompanies this letter, and which has enabled myself and Mr. Girdwood to detect that fearful poison in the blood, liver, tissues, and contents of stomachs of animals poisoned by doses such as those Dr. Taylor administered in the experiments on which he founded his theory promulgated at the late trial, and which has also enabled us to separate the strychnine from the tissues and organs of a dog after the body had been interred twelve months. The results of these experiments, but without a description of the process employed, were forwarded by myself and Mr. Girdwood for the consideration of Sir George Grey, as we were of opinion that if John Parsons Cook was poisoned by strychnine, no matter how small the fatal dose, its presence could even now be clearly demonstrated if the tissues of his body were subjected to the same mode of analysis.

"I cannot conclude this letter without expressing my opinion that, as of all known poisons there is not one more readily detected in the tissues than strychnine, consequently no death ought to be attributed to its agency unless its presence be clearly demonstrated."

"The process alluded to in the above is as follows:—The tissues of the body are rubbed with distilled water in a mortar to a pulp, and then digested, after the addition of a little hydrochloric acid, in an evaporating basin; then strained, and evaporated to dryness over a water-bath; digest the residue in spirit, filter, and again evaporate to dryness; treat with distilled water, acidulated with a few drops of hydrochloric acid, and filter; add excess of ammonia, and agitate in a tube with chloroform; the strychnine in an impure condition is entirely separated with the chloroform. This chloroform solution is to be carefully separated by a pipette, and poured into a small dish, and evaporated to dryness; the residue is moistened with concentrated sulphuric acid, and heated over a water-bath for half an hour; water is then added, and excess of ammonia—again agitated with chloroform, and the strychnine will be again separated by the chloroform, now in a state of sufficient purity for testing, which can be done by evaporating a few drops on a piece of white porcelain, adding a drop of strong sulphuric acid, a minute crystal of bichromate of potash being added in the usual way; or the elegant mode proposed by Dr. Letheby can be adopted."

That strychnine *was* either in the contents of the stomach or in some of the tissues of Cook's body, able chemists entertain no shadow of a doubt. Had Taylor been happily successful in his analysis, and had detected even the 50,000th part of a grain of the poison, that discovery, conjoined with the overwhelming and crushing circumstantial evidence of Palmer's guilt, would have settled his conviction and condemnation in a few hours.

As to what was the real cause of Cook's death, Brodie, Todd, and others have no doubt. They affirm that it was strychnine. The questions raised by the defence of the possibility of Cook's death being the result of some tetanic disease, instead of poison, is alleged to have broken down, and disappear in the course of the cross-examination by the Attorney-General.* It has subsequently been conjectured that death might possibly have arisen from some new form of disease, of a tetanic character, not yet recognised.† Cook was said to have died with identically the same symptoms, even to the very last expression of "Turn me over," as Mrs. Sargeantson Smyth and Mrs. Dove, in both of whose cases strychnine was known to have been the cause of death beyond dispute. Five theories were set up by the defence in opposition to the fact of the identity of death from strychnine in Cook's, Dove's, and Smyth's cases. The five theories were—idiopathic and traumatic tetanus, tetanic complications, epilepsy, and angina pectoris.

As to idiopathic tetanus, it was asked, where were the signs

* "It is not, of course, for us to suggest what considerations may have acted with greater force upon the minds of the jury, but we should have thought the medical symptoms, and the manner of their succession, quite conclusive. It was proved by such an agreement of medical testimony as we do not remember to have seen in any former case, that the symptoms observed in the case of John Parsons Cook were inconsistent with any other hypothesis than that of death caused by poisoning by strychnine. The attempts made for the defence to neutralize this authoritative and direct testimony resulted in an entire failure. We had enough of such miserable jargon as 'epileptic convulsions with tetanic complications;' but we put it to the recollection of any person who was present at the trial, or who has read the reports of it with ordinary attention, if the overpowering weight of credible medical testimony was not in favour of the view that such symptoms as those of Cook were inconsistent with any other hypothesis than that of death by strychnine?" —*The Times*, June 5, 1856.

† "Only one question was unconsidered in this trial, and that question, though among the bare possibilities, is so highly improbable that it was perhaps well not to raise it. Cook's symptoms were not the symptoms of any known disease. But within this century has not a disease before unknown been generated in India, and propagated throughout the world? Forty years ago the symptoms of Asiatic cholera were not the symptoms of any known disease, and would have been confidently attributed to poison. Indeed, the ignorant everywhere imputed the ravages of the disease to poison. Experience has corrected the error; but the question to be asked is, what must have been thought of the first case or cases, what mistakes made about its nature unknown? A new disease is then among the possibilities; and it might have been fitting to consider the question, if the circumstances of the case, as well as the character of the symptoms, had not so strongly borne out the conclusion of death by the strychnine procured and substituted by Palmer for the medicine supplied by Mr. Bamford."—*The Examiner*, May 31, 1856.

of it? If it was traumatic, where was the wound or injury of a nerve to account for it? No one could point it out. As for tetanic complications, the witnesses for the Crown scouted the idea. Epilepsy—Was it epilepsy? Not one of the medical witnesses either for the prosecution or the defence could say it was. Hydrophobia would have been a much more plausible theory to account for Cook's symptoms—though it does not appear that Cook had ever been bitten by a mad dog—than the suggested one of angina pectoris; for hydrophobia *is* a tetanic disease, whereas angina pectoris *is not.**

One question more remains to be considered,—viz., whether, if chemistry fail to discover any poison in the corpse, it is proof absolute or presumptive of no poison having been administered? Here, again, we apprehend that chemistry is inadequate to the task of deciding so vital a question, and that the physician alone is the referee whose testimony can be relied on in explaining the matter. For different persons are susceptible of the operation of the same medicine in different degrees, according to the peculiar idiosyncrasy of the patient; one person being killed by a dose of poison so minute, that it would be innoxious to another, and *vice versa.* Every medical man knows the susceptibility and insusceptibility of particular patients to the action of particular remedies,—as of mercury, for instance. Thus, half a grain will salivate one patient, while large and frequently-repeated doses will scarcely affect the system of another; nor is it possible to foresee or explain these peculiarities. Here, then, is a set of cases in which chemistry is likely to be greatly at fault, but where the practical physician can alone determine whether the symptoms at the time of death were those of poison, be the dose large or small, and whether the particular symptoms were those of this or that kind of poison, though no poison be subsequently detected in the organic remains. We are, therefore, forced to conclude that chemistry is subsidiary to medicine, and that analytic chemistry is overrated when its *ipse dixit* is received as

* The analogy between traumatic tetanus and hydrophobia is much closer and stronger than at first sight appears probable. Traumatic tetanus, or trismus, arises from the injury of a nerve, generally of the toe or thumb. Hydrophobia arises from the bite of a dog or cat, rabid or not, as it may be; for it is not absolutely proved that the animal must needs be rabid. But, by the bite, the cutaneous nerves are lacerated or punctured in a peculiar way. Now, the first symptom is that of shuddering, and a sense of creeping over the skin, aggravated by a light touch, or a current of cold air. Then the sight of any liquid in motion produces the horripilations of the skin. After this, the back of the head and spine become painful; and, lastly, the difficulty of swallowing ensues, and is the same as the lockjaw, or trismus, being a reflex act from irritation of the top of the spinal chord and medulla oblongata. Both in hydrophobia and lockjaw the intellect may remain entire to the last.

the ultimatum of an inquiry in a court of law, and its testimony allowed alone to determine the guilt or innocence of the accused person. We have already declared our conviction that the verdict in Palmer's case did not turn upon the chemical, but upon the moral or circumstantial evidence; and we have followed out this train of argument for the express purpose of drawing the true line of demarcation between chemistry and medicine, and of placing each of them on its proper basis. A great deal of the confusion of ideas would have been saved on the recent occasion, had there been a Crown officer of anatomy to perform the *post-mortem* examination, of chemistry for the chemical experiments, and of medicine for the medical symptoms, independent of the scientific witnesses subpœnaed on either side, for the elucidation of the truth; and the appointment of such officers is not more preposterous than the law-officers of the Crown, without whom justice could never be awarded.

We subjoin from the *Times* a truthful and graphic account of this wretched criminal's execution. The day previously, Mr. Smith, his solicitor, was summoned by telegraph from London to Stafford, at Palmer's earnest request, and he arrived at half-past 10 o'clock at night, and had an interview with the convict, in the presence of Major Fulford, the governor of the gaol. The prisoner had declined to retire to rest until Smith came, and from that circumstance and the anxiety he had shown to have him sent for, it was supposed that he had some important communication to make to him; but it was not so. On going into the cell, the Governor informed Palmer that if he had anything confidential to say on family affairs to Mr. Smith, he (the Governor) would keep it a secret. The prisoner replied that he had not, and he hoped the Governor would lose no time in publishing all he said. He also added, all he had to say was to thank Mr. Smith for his great exertions—the officers of the prison for their kindness to him—*and that Cook did not die from strychnine. Major Fulford expressed a hope that in his then awful condition he was not quibbling with the question, and urged him to say "Ay" or "No," whether or not he murdered Cook. He answered immediately, "Lord Campbell summed up in favour of poisoning by strychnine." The Governor retorted, it was of no importance how the deed was done, and asked him to say "Yes" or "No" to the question. Palmer said, "he had nothing more to add. He was quite easy in his conscience, and happy in his mind."* This is the Governor's version of the conversation; but upon the material point Mr. Smith stated, just after leaving the convict, that what Palmer said to him was, *"I am innocent of poisoning Cook by strychnine;*

*and all I ask is, that you will have his body examined, and that you will see to my mother and boy."*

Towards midnight he had taken a painful leave of his immediate relatives, and was now awaiting his irrevocable doom. He had slept two hours and a half in the early morning, and, on awakening, the Chaplain entered his cell. He said, in reply to a question asked by the rev. gentleman, that he felt comfortable, and was quite prepared. He continued in bed until half-past 5, when he had some tea, and again at half-past 7. To the warder who brought him the tea on the last occasion, and who asked how he was, he said he was very comfortable. The Chaplain remained in almost constant attendance upon him until the hour of his execution. Shortly after 7 o'clock, Lieutenant-Colonel Dyott, the high-sheriff, and Mr. Hand, the under-sheriff, arrived at the gaol, and at once proceeded to the prisoner's cell, where they found him in earnest conversation with the Chaplain.

After a brief interval, the High-Sheriff asked him if he was prepared to admit the justice of his sentence. He replied, with the most energetic gesticulation, "No, sir, I do not; and I go to the scaffold a murdered man." He added that several persons, whose names he would not mention, were guilty of his murder, and that he could not acknowledge the justice of his sentence. The cell of the prisoner was one of a series situate on the first floor of an oblong building, around which a light iron gallery was thrown. Almost immediately opposite the door of his cell a bridge went across to the gallery on the opposite side, and from the centre of the bridge an ornamental stair of iron descended into a large corridor on the basement story. Here were stationed, shortly before 8 o'clock, the High-Sheriff of the county and the Under-Sheriff; Mr. W. H. Chetwynd, a magistrate of the county; Major Fulford, the governor of the gaol; Mr. Hatton, the chief of the county constabulary, and the representatives of the press, awaiting the awful ceremony about to take place. At that moment, a tall, broad-shouldered, elderly man, with short grey hair, and dressed in a white smockfrock, emerged from a room in the corridor, and ascending the light iron stairs, entered the condemned cell. This was the executioner, a labouring man residing at Dudley, named John Smith, and this was his first introduction to the convict, whom he at once proceeded to pinion in the presence of the High-Sheriff and the Chaplain. While this operation was being performed Palmer betrayed no symptom of emotion, and simply requested that the cord might not be drawn too tightly. The High-Sheriff and the Chaplain then left the cell for a short time, and the prisoner remarked to the officers who attended him that they would observe that he had not changed from what he had always said, and he then said, "All I have to ask of you is to pray for my child." The High-Sheriff and Chaplain again visited the cell, and, thinking that the prisoner might perhaps object to say anything in the presence of the officers, they were requested to withdraw. At this moment all the preparations were complete. The unhappy man was pinioned, the executioner was standing by him, and nothing was required but the signal to move forward to the scaffold. The Chaplain, in the most solemn manner, exhorted him to admit the justice of his sentence. The prisoner firmly replied that it was not a just sentence. "Then," said the Chaplain, "your blood be upon your own head." To this observation the prisoner made no answer.

At this moment the prisoner appeared for an instant at the door of his cell, and took a cursory look at the official gentlemen waiting below to conduct him to the scaffold. He entered his cell again, and immediately afterwards the Chaplain and the High-Sheriff emerged from it, accompanied by the convict, who tripped nimbly down the stairs into the corridor, followed by the executioner. The remarkable appearance of the prisoner at this time will not easily be forgotten. Contrary to all usage, he wore the prison dress, consisting of a dark grey jacket, trousers and waistcoat, all of the coarsest description, a blue checked cotton shirt, and a pair of thick list shoes. He carried a handkerchief in one hand, of the same coarse material. At his own request, his light sandy hair had been closely cropped, which brought the whole configuration of his large round head and face into striking prominence, and, with the dress he wore, gave to his whole *physique* an air of singular repulsiveness which was not at all natural to him. It ought, however, to be stated that the wearing the prison dress was not intended as an indignity, but simply arose from the circumstance of his having no clothes of his own in the prison. The melancholy procession was now formed which was to conduct him to his doom. The Chaplain went first, reading the burial service, followed by the Under-Sheriff, then by the High-Sheriff, carrying their wands of office, next by Palmer, then by the executioner, and finally by Major Fulford, the governor of the prison, Mr. Hatton, the chief constable, and several of the officers of the gaol; and in this way he was escorted to the scaffold amid the tolling of the prison bell. His bearing in these last moments of his life elicited the amazement of all who witnessed it. As he passed Major Fulford, who was waiting to fall into the procession, he bowed to him in an easy, offhand manner, and then stopped for an instant to shake hands with one of the officials of the prison whom he recognized.

He marched along with a light, jaunty step; but the expression of his mouth and the pallor with which his features were suffused indicated the deep current of natural emotion which he strove in vain to conceal. The distance he had to traverse from his cell to the scaffold was very considerable, and included three short flights of stairs, but his step never for an instant faltered. As the procession reached the entrance of the prison, Mr. Wright, the philanthropist, who was standing near, stepped back to allow it to pass; the convict bowed courteously to him, and then walked lightly up the steps leading to the scaffold, and of his own accord placed himself under the beam. The executioner at once proceeded to adjust the rope round the culprit's neck, and was about to retire from the scaffold, when he seemed to remember that he had not drawn the white cap over his face. He returned to do so, and then the convict shook hands with him and bade him good-bye. An instant elapsed before the bolt was withdrawn, and the rapid inflation and collapsing of the part of the cap which covered his mouth evinced the intensity of his feelings at this awful moment. The drop at length fell, and he died almost without a struggle. Once or twice, when the executioner was gently holding down his legs, he raised himself slightly up, and there was a simultaneous convulsive movement of the shoulders for an instant; but he exhibited no other sign of life. He held a handkerchief in one of his hands, where it still remained tightly clenched when the body was cut down. With some very slight exceptions, the deportment of the crowd, among whom were many decently-dressed women, was decorous in the extreme.

So ended the life of William Palmer. It will be perceived that he made no confession of his crime.* This is, on public grounds, deeply to be lamented and deplored. When pressed to confess, all that he would admit was, that "Cook did not die from strychnine." It was obvious, by his refusing to answer the question repeatedly and earnestly addressed to him a few minutes before his execution, whether he was instrumental in Cook's death, that he was exercising some mental reservation on the

* "On the morning before his execution, he asked the Rev. Mr. Sneyd if a sinner could be saved who confessed to God, but preserved silence towards men. The reverend gentleman declined to give a positive answer, lest he should be thought to encroach upon the divine prerogative of mercy. But after further self-deliberation, he returned to the prisoner's cell, and said to him, 'You have asked me a difficult and abstract question. Your Bible tells you that all liars shall have their part in the lake of fire and brimstone. If you persist in proclaiming your innocence when you know that you are guilty, you will die with a lie in your mouth, and you know the consequences.' The tears stood for a moment in Palmer's eyes, but he quickly recovered himself, and made no further remark."—*The Leader.*

point. When Major Fulford begged him to admit the justice of his sentence and unburthen his conscience before entering into the presence of his Maker, Palmer's remark was, "Cook did not die from strychnine;" and when implored to say "Yes" or "No" to the question—was he not the murderer of Cook, he replied, "*I have nothing more to add; Lord Campbell summed up for strychnine.*" If Palmer had positively repudiated all participation in Cook's death, his denial of the fact, even at the awful moment immediately preceding his execution, would not have been entitled to one moment's consideration, or to the slightest credence; but, as Palmer would not deny his guilt, but persevered to the last in emphatically asserting that Cook did not die from the effects of strychnine poison, we think we are justified, according to the recognized rules of evidence, in concluding *that strychnine was not the specific poison that caused Cook's death.* The reader must view Palmer's statement not only in conjunction with the fact that Drs. Taylor and Rees could not discover strychnine in the contents of Cook's stomach, but in relation with the conflicting medical testimony adduced at the trial, as to the true character of the symptoms exhibited by Cook during his last fatal illness. We cannot conceive how any person, accustomed to consider and weigh nice points of evidence, can arrive at any other conclusion. That Palmer murdered Cook is, to our mind, an indisputable fact; *but, according to our apprehension, strychnine was not the poison used for the purpose!* Well, then, it may be asked, if Palmer was conscious of his having accomplished his murderous designs by the administration of some other poison, and not by strychnine, how can his solemn declaration—"I am an innocent man"—be made consistent with such an hypothesis? It must be borne in mind that William Palmer was indicted for murdering John Parsons Cook *by means of strychnine.* He was accused, tried, convicted, sentenced, and hanged for committing the murder *in the manner set forth in the indictment.* If strychnine had nothing to do with Cook's death,—if the poison had never been exhibited by Palmer to Cook, or by any other person with his knowledge,—then Palmer was falsely convicted, *for he was innocent of the particular offence imputed to him.* If a man is accused, tried, convicted, and hanged for drowning a person found, under questionable and suspicious circumstances, dead in the water, and he had no more hand *in so destroying him* than the Emperor of China or the King of the Sandwich Islands, the accused party is wrongly convicted and unjustly punished. He may, some days prior to the death of the party found drowned, have administered to him some deadly drug, or have given him a blow on the head, thus causing temporary mental derangement, impelling the party to the act of suicide, but of the particular and specific offence for which he is tried, convicted, and executed—viz., murder by drowning—he is clearly and undoubtedly innocent. The fact of the man being a murderer and justly deserving death upon the scaffold does not affect our position. The offence or crime for which a party is arraigned must be clearly established against him before he can legally be found guilty and punished. There would be no safety or security for society unless this principle were strictly, stringently, and jealously adhered to in the administration of the criminal jurisprudence of the country. We feel anxious to place this question fairly, dispassionately, and legitimately before our readers, not having the faintest shadow of a doubt as to the guilt of the miserable man who has gone to his last account, or as to the *moral* justice of his conviction, sentence, and death.

We are of opinion that the important point under consideration should not be permitted to rest unsolved. If a mistake has been committed, no harm can arise from a candid and honest acknowledgment of the fact. If an exhumation of the body of Cook should take place, and the discovery of strychnine or some other poison in the tissues be detected, then much dissatisfaction would be removed from the public mind. It is useless to close our eyes to the fact that such a feeling exists to a great extent in this country, and among those, too, who were most profoundly prejudiced against the unhappy man, and who called loudly for the execution of the law. We subjoin, in conclusion, the following able remarks of a contemporary in reference to Palmer's last moments.

"The hope that Palmer in his last moments would make such a confession as would clear up once and for ever the doubts that still remain in the minds of many persons as to his guilt, has been disappointed; and his last words will, we fear, only tend to strengthen the opinion of those who denied that Mr. Cook died from the effect of strychnine. The few words that the wretched criminal uttered before proceeding to the scaffold, instead of clearing up the difficulties which have beset the minds of many medical men, will only tend to still further perplex them. 'I am innocent of poisoning Cook by strychnine,' was all that Palmer, whilst standing upon the verge of eternity, could be brought to say, although urged in the most solemn manner to admit or deny the fact of his having *murdered* Cook. The general impression seems to be that this denial was a mere quibble, and that he 'paltered with us in a double sense' in denying the justice of his sentence merely because 'Lord

Campbell summed up in favour of strychnine.' It seems to us, however, that if we admit the possibility of a man quibbling and attempting to deceive with the halter round his neck, he would not much fear going into the presence of his God with a lie in his mouth; we therefore do not attach much importance to his denial of having given Cook this poison. It may be, however, that some drug was used in the pills substituted for those ordered by Dr. Bamford, which Palmer affected to believe was the cause of death rather than the strychnine.

"The correspondence which has lately taken place between Mr. Herapath and Sir Fitzroy Kelly, relative to Tawell's case, would seem to show that the pseudo-Quaker's confession was in some respects similar to the statement made by Palmer. 'It is true,' says Sir Fitzroy Kelly, 'that he (Tawell) confessed his guilt; but I have the best reason to believe that his confession was that he committed the murder, indeed, but in a manner totally inconsistent with the truth of the scientific evidence upon which he had been convicted.' We can easily understand that a man, in order to escape as far as possible the posthumous infamy which attaches to the memory of a diabolical murderer, may so colour certain facts known only to himself, as to lead him, not perhaps wholly in bad faith, to deny the legal justice of his sentence. This is the only manner, however, in which we can excuse him from the horrible crime of telling what we cannot help thinking to be a fearful falsehood, whilst just *without* the presence of the Almighty, if we may be allowed to use such a phrase when speaking of the Omnipresent.

"Although it may appear late, we reiterate our desire that the body may be once more exhumed for the purpose of thoroughly examining every portion of it in the presence of the leading toxicologists of the day. It will be said, perhaps, that such a course would seem to imply a doubt that Palmer was really guilty—a fearful doubt even to suggest, now that it is beyond the power of man to see justice done to him. We do not think, however, that any such objection should stand in the way of clearing up the case, as far as it can be cleared up indeed, by a more thorough and patient examination of the body than it has yet received. Indeed, we think the very equivocation of the murderer on the scaffold might be used as an argument for the course we recommend. Taking for granted that Cook was poisoned by Palmer, as the whole country most undoubtedly does, and taking for granted, as many people do, perhaps, that Palmer was speaking the truth in denying that he poisoned Cook with strychnine, the question which arises next is, With what was the murder committed? What subtile agent could have been employed that killed, and yet escaped the notice of men of science? It seems to us that not only those who believe strychnine was the agent used by Palmer should desire a further analysation of the subject, but also those who deny it, and yet believe that he drugged his victim to death."*

---

### Part Second.

### Reviews.

### ON SUICIDE AND SUICIDAL INSANITY.†

* "The Association Journal," 21st June.

